# The Effect of Lifestyle on the Quality of Life of Vulvar Cancer Survivors

**DOI:** 10.3390/cancers17061024

**Published:** 2025-03-18

**Authors:** Marleen S. Boonstra, Anke Smits, Viktor Cassar, Ruud L. M. Bekkers, Yvonne Anderson, Nithya Ratnavelu, Tineke F. M. Vergeldt

**Affiliations:** 1 Northern Gynaecological Oncology Centre, Queen Elizabeth Hospital, Gateshead NE9 6SX, UK; marleen.boonstra@ru.nl (M.S.B.); viktor-ivan.cassar.1@gov.mt (V.C.); yvonne.anderson6@nhs.net (Y.A.); nithya.ratnavelu@nhs.net (N.R.); tineke.vergeldt@nhs.net (T.F.M.V.); 2 Faculty of Medical Sciences, Radboud University, 6525 Nijmegen, The Netherlands; 3 Department Obstetrics and Gynaecology, Radboudumc, 6525 Nijmegen, The Netherlands; ruud.bekkers@radboudumc.nl; 4 Department of Gynaecology, Catharina Hospital Eindhoven, 5602 Eindhoven, The Netherlands; 5 GROW School for Oncology and Reproduction, Maastricht University, 5623 Maastricht, The Netherlands

**Keywords:** vulvar cancer, gynecological cancer, quality of life, lifestyle, BMI, survivorship

## Abstract

**Introduction**: Vulvar cancer affects approximately 47,000 women annually worldwide. With most studies focusing on oncological outcomes, quality of life is often overlooked. There is a lack of knowledge on the influence of modifiable factors such as lifestyle on the quality of life of vulvar cancer survivors. This study evaluated the association between lifestyle factors and the quality of life of vulvar cancer survivors. **Methods**: This was a cross-sectional survey study of women who received surgical treatment for vulvar cancer ≥FIGO stage 1B at the Northern Gynecological Oncology Centre, UK, between 2013 and 2022. Baseline and clinical characteristics were collected from patient records. Godin Leisure-Time Exercise questionnaires were used to assess physical activity. BMI was assessed using self-reported height and weight. Quality of life was measured using the validated European Organization for Research and Treatment of Cancer Core Quality of Life Questionnaire (EORTC QLQ-C30) and the vulvar cancer-specific module (VU-34). An analysis was performed using Mann–Whitney-U and Kruskal–Wallis tests. **Results**: Of the 299 women, 139 were eligible for participation, of whom 58 participated (41.7%). Twenty participants had a sedentary (40.8%), eight a moderately active (16.3%), and seventeen an active (34.7%) lifestyle. Active participants reported higher overall quality of life and higher functioning in all domains but not for vulvar-related symptoms or sexual functioning. Forty-nine participants disclosed their BMI, which was not associated with quality of life outcomes. **Conclusions**: A higher level of physical activity was associated with higher quality of life. No association was found between BMI and quality of life.

## Simple Summary

Vulvar cancer affects approximately 47,000 women per year worldwide. As treatment options have improved, the number of survivors is increasing, and with that, the focus on their quality of life. This study evaluated the effect of physical activity and body mass index (BMI) on the quality of life of vulvar cancer survivors. Women who had been treated with surgery for vulvar cancer stage IB or higher were asked to participate. We found that a better physical activity level was associated with a better quality of life. Interestingly, BMI was not associated with the quality of life of survivors. We, therefore, conclude that more attention needs to be given to the role of physical activity during the survivorship era of vulvar cancer patients to improve their quality of life.

## 1. Introduction

Gynecological cancers are amongst the most common cancers to affect women, with vulvar cancer being diagnosed in about 47,000 women worldwide every year [1]. Vulvar cancer is defined by atypical cell growth on the external genitals and typically arises between the ages of 50 and 80, with the Human Papilloma Virus (HPV) and lichen sclerosis being common risk factors [2]. Around 39% of vulvar cancer cases are associated with HPV, and around 2–4% of women with vulvar lichen sclerosis develop an invasive carcinoma within 10 years of diagnosis [3,4].

Treatment usually consists of surgery of the vulva and removal of groin nodes, possibly followed by additional (chemo)radiotherapy [5]. Unfortunately, surgical treatment is associated with a high risk of complications such as wound infections and breakdown, which has been reported in 30% to 85% of women [6,7]. In addition, women are prone to develop long-term sequelae such as lymphoedema after groin node surgery, which occurs in 25% of women [8]. Survivors also often struggle with their body image and self-confidence and report a reduction in sexual well-being [9]. A study by Alimena et al. reported that one-third of women were afraid to have sex and over 50% did not like the appearance of their body [10]. Due to advancements in treatment, survival has improved, leading to an increase in survivors and quality of life increasingly being recognized as an important outcome. However, little is known about the post-treatment quality of life of vulvar cancer survivors.

Previous studies in other gynecological cancers, including endometrial and ovarian cancer, have suggested that women report a reduced quality of life in the years after treatment [11]. Despite quality of life being a multidimensional evaluation, influenced by more than diagnosis and treatment alone, there is a clear relationship between an unhealthy lifestyle and quality of life, with obesity and a sedentary lifestyle being associated with a poorer quality of life [12]. Currently, more than half of the women with vulvar cancer are overweight and 26% are obese [13,14]. In addition, studies have reported that around one-third of women with vulvar cancer have a sedentary lifestyle [15]. However, despite the growing number of survivors with an unhealthy lifestyle, studies evaluating its effect on the quality of life for this group of women are scarce.

With this study, we aim to assess the association between lifestyle factors such as physical activity and body mass index (BMI) and the quality of life of vulvar cancer survivors. Through this study, we hope to gain a better understanding of the variability of their quality of life and tailor women’s care to improve quality of life.

## 2. Methods

### 2.1. Study Design

A cross-sectional survey study was performed on women who were surgically treated for vulvar cancer between January 2013 and December 2022 at the Northern Gynecological Oncology Centre (NGOC), Gateshead, United Kingdom. Women diagnosed with squamous cell carcinoma of the vulva were identified through the regional database in North East England and Cumbria. We included women with at least stage IB disease, as classified by the International Federation of Gynecology and Obstetrics (FIGO) [16]. Stage IA was excluded as treatment is less invasive due to the small tumor size and absence of lymph node surgery. Type IA can also be treated by general gynecologists; therefore, data on these patients were not always present. Women who were unable to complete the questionnaires due to cognitive impairment or who were unable to communicate in English were excluded from the study. Eligible women were individually approached to participate. The project was a service improvement audit for vulvar cancer care and was, therefore, exempt from ethical review.

### 2.2. Data Collection

Patient data were obtained from electronic patient records. These data included age at diagnosis, alcohol consumption, smoking status, BMI at time of diagnosis, American Society of Anesthesiologists score (ASA), comorbidities, histology of the tumor, FIGO stage, treatment details, and disease recurrence. BMI was also calculated using women’s self-reported height and weight at the time of the survey. BMI < 30 kg/m^2^ was defined as non-obese and BMI ≥ 30 kg/m^2^ was defined as obese according to UK national guidelines [17]. The Godin Leisure-Time Exercise questionnaire measured the level of physical activity on a weekly basis. Participants were asked to report the number of times they participate in ≥15 min of strenuous, moderate, and mild activity per week. Leisure Score Index (LSI) values were calculated, and participants were assigned as sedentary/insufficiently active (LSI < 18), moderately active (LSI 18–24), or active (LSI > 24) [18].

Women were contacted for participation by telephone and by regular mail. They received a paper version of the questionnaire at home or a link to the online version, depending on their preference. A follow-up telephone call was performed if completed questionnaires had not been returned. Women who could not be reached by telephone were sent the questionnaires by regular mail.

### 2.3. Outcomes Measures

The validated European Organization for Research and Treatment of Cancer (EORTC) Core Quality of Life Questionnaire (QLQ-C30) and the vulvar cancer-specific module (VU-34) were used to assess quality of life and symptoms after vulvar cancer treatment [18,19]. The EORTC-C30 consists of 30 cancer-specific questions that cover several domains within quality of life: emotional, physical, cognitive, and social functioning. It also measures symptoms and overall quality of life. The EORTC questions were rated on a 4-point Likert scale from 1 (“not at all”) to 4 (“very much”). Overall health and overall quality of life were rated on a 7-point Likert scale, ranging from 1, being “very poor”, to 7, being “excellent”. The vulvar cancer module further evaluates sexual functioning and symptoms specific to vulvar cancer treatment. Questions on bladder, bowel, and sexual functioning were dependent questions on the presence of urine or bowel stomas and sexual activity. A higher score for quality of life questions indicated a higher quality of life and functioning, and a higher score for symptoms related questions indicated higher symptomatology. A higher score for the Godin Leisure-Time Exercise questionnaire indicated a more active lifestyle.

### 2.4. Data Analysis

Data were analyzed using IBM SPSS statistics version 29.0 [20]. The EORTC-C30, VU-34 and Godin Leisure-Time Exercise questionnaires were scored according to their scoring manual [18,21].

Medians with interquartile ranges (IQRs) were used to describe non-normally distributed continuous variables, while means and standard deviations (SDs) were used to describe normally distributed variables. Frequencies and proportions were used to describe categorical variables. Independent samples *t*-tests and non-parametric tests (Mann–Whitney U test, Kruskal–Wallis test), Pearson chi-squared tests, and Fisher’s exact tests were used accordingly, depending on the type of data. Multivariate analyses were performed as appropriate, using ANOVA to correct for age, ASA, FIGO stage, and primary vulvar surgery as confounding factors. Two-sided tests were used, with *p*-values under 0.05 being regarded as statistically significant. 

## 3. Results

**Figure 1:**
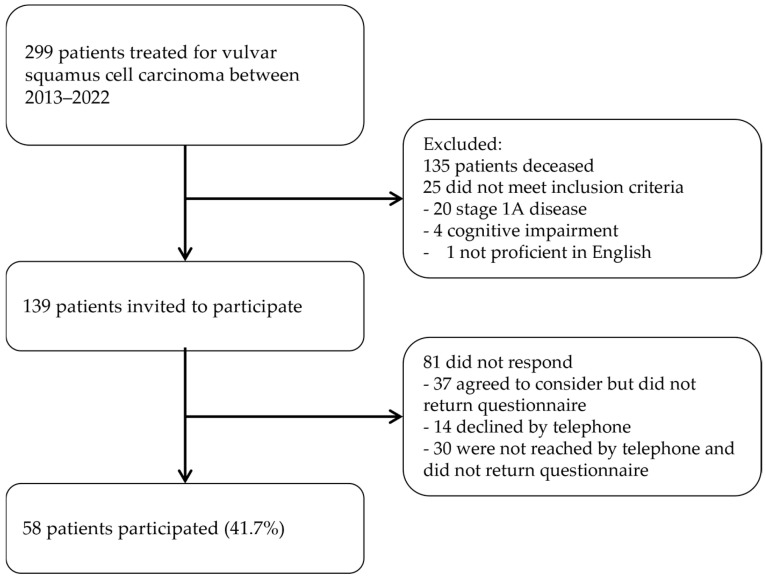
Flowchart of inclusion process.

In total, 299 women were surgically treated for vulvar squamous cell carcinoma FIGO stage ≥ IB at the NGOC between January 2013 and December 2022. Of these, 135 (45%) were deceased at the time of the study. A further 25 women were excluded because they did not meet the inclusion criteria (Figure 1). Cognitive impairment was present in four women, and one woman was not proficient in English. A total of 139 women were eligible and invited to take part in the survey. Of those, 58 women participated by completing the questionnaires, resulting in a response rate of 41.7%.

The baseline characteristics of respondents and non-respondents are summarized in Table 1. The average follow-up was 64.6 months (IQR 31.0–95.0) for the respondents and 70.1 (IQR 34–96) for non-respondents. For participants, the mean age was 68 (SD 10.7) and median BMI was 28.5 (IQR 24.4–32.9). The majority of women were diagnosed with FIGO stage IB disease (74.1%) and had received groin node surgery as part of their treatment (89.7%). They were most often classified as ASA score 2 (41.4%). Half of the women had a background of lichen sclerosis. When comparing participants and non-participants, there was a significant difference seen in age, with respondents being significantly older than non-respondents (*p* < 0.001). There were no significant differences in other clinical characteristics.

The results of the EORTC-C30 and VU-34 questionnaires are shown in Table 2. Out of the 58 women who completed the questionnaires, 49 disclosed their current height and weight, and 32 were categorized as non-obese (BMI < 30 kg/m^2^) and 17 as obese (BMI ≥ 30 kg/m^2^). Nine women did not disclose their height and/or weight, of whom four were obese, three were overweight, one had normal weight and one had an unknown BMI at the time of their diagnosis.

The participants with BMI ≥ 30 kg/m^2^ reported a global health score of 66.7 (IQR 66.7–83.3) and the non-obese group reported a score of 62.5 (IQR 50.0–83.3), which was not significantly different (*p* = 0.426). None of the functioning scales were significantly different between the BMI groups (Table 2). Fatigue and insomnia were the most prevalent reported symptoms. There were no differences in reported symptoms between the groups. When looking at vulvar-specific symptoms, most reported symptoms were vulvar scarring, leg lymphoedema, and urine urgency and leakage. The non-obese group reported a median score of 8.3 (IQR 0.0–29.2) for leg lymphoedema, while the obese group reported a median score of 50.0 (IQR 16.7–75.0). This was significantly different at univariate analysis (*p* = 0.006); however, this did not remain significant after adjusting for confounders, including age, ASA, FIGO stage, and primary vulvar surgery (*p* = 0.155). Body image was reported as 77.8 (IQR 56.0–100) and 44.0 (IQR 22.2–66.7) for the non-obese and obese groups, respectively (*p* = 0.043), but this also did not remain significant after multivariate analyses (*p* = 0.132).

The Godin Leisure-Time Exercise questionnaire, which was completed by 49 participants, showed that 40.8% of participants (N = 20) had a sedentary, 16.3% (N = 8) a moderately active, and 34.7% (N = 17) an active lifestyle. Four participants (8.2%) who disclosed their height and weight, did not disclose their physical activity levels; hence, only 45 participants could be included to assess the association between BMI and physical activity levels. The median activity score was 21 (IQR 6–36) for the non-obese group, and the median activity score was 13.5 (IQR 6–30) for the obese group (*p* = 0.824). There was no significant difference seen between physical activity levels in both groups (*p* = 0.839) (Table 3).

The quality of life outcomes across the different physical activity groups are shown in Table 4. Active participants reported a median global health score of 83.3. This was significantly higher than the sedentary group and the moderately active group with reported scores of 50.0 and 75.0, respectively (*p* ≤ 0.001). Additionally, increased activity levels were significantly associated with a higher score for all functioning scores (Table 4). The greatest differences were seen for physical functioning, role functioning, and emotional functioning, with respective median scores of 100 for these three domains in the active group. This is in contrast to the moderately active group, who reported scores of 81.7, 83.3, and 70.8, as well as the sedentary group, who reported scores of 60.0, 50.0, and 66.7, respectively. These differences all remained statistically significant after adjusting for confounders including age, ASA, FIGO stage, and primary vulvar surgery. The active group reported significantly lower scores for fatigue (*p* ≤ 0.001), nausea and vomiting (*p* = 0.010), insomnia (*p* = 0.017), and appetite loss (*p* = 0.002). These differences remained significant after adjusting for age, primary vulvar surgery, and FIGO stage and ASA score, except for nausea and vomiting (*p* = 0.068) and insomnia (*p* = 0.053). No further significant differences were seen for the vulva-specific module.

## 4. Discussion

This study aimed to assess the association between physical activity, BMI, and quality of life of vulvar cancer survivors over a 10-year period at the NGOC. We found that physical activity was associated with higher self-reported quality of life in all domains but was not associated with symptom distress. In addition, we did not find an association between BMI and quality of life or symptom distress.

This study is one of the first studies to assess the association between the physical activity, BMI, and quality of life of vulvar cancer survivors and the impact of cancer and surgery on quality of life. Unfortunately, quality of life is still an often overlooked part of survivorship care after vulvar cancer, and the literature remains scarce for this population. Studies on other gynecological cancers concur with our findings, stating that higher physical activity levels are associated with better quality of life [22,23,24]. A prospective study by Fleming et al. of 408 gynecological cancer patients up to 2 years after diagnosis, including 22 patients with vulvar/vaginal cancer, showed that physically active survivors reported the highest overall quality of life scores, and that an increase in physical activity led to an increase in measured quality of life [24]. Similarly, Zainordin et al. reported that 76.8% of breast and gynecological cancer survivors were insufficiently active and that these patients experienced poorer physical functioning, more insomnia, and more constipation [25]. Furthermore, the review of Babatunde et al. shows that endometrial cancer survivors’ inactivity is significantly correlated with poorer overall quality of life [23]. Several studies in ovarian cancer confirm these findings, with physical activity levels being specifically linked with functioning and symptomatic quality of life domains [22,26]. Our study was of a cross-sectional nature and, therefore, was not able to make statements about changes in the activity levels of vulvar cancer survivors. In our study, we did not find any association between physical activity levels and vulva-specific symptoms including lymphoedema. This is contradictory to reports in other cancer areas, where they show that lower physical activity was significantly associated with lower limb lymphoedema [27,28]. This may be a result of our sample size or the duration of follow-up.

In our study, we did not find an association between BMI and quality of life, which is not in line with the previous studies of gynecological cancers, which reported that higher BMI is significantly associated with a poorer quality of life [12,29]. A systematic review by Smits et al. showed that obese endometrial cancer survivors (BMI > 30 kg/m^2^) had significantly poorer reported quality of life, including poorer physical, social, and role functioning compared to non-obese survivors (BMI < 30 kg/m^2^) [30]. In addition, Smits et al. also reported a significantly poorer self-reported quality of life in obese ovarian cancer survivors, as well as worse physical functioning for women with a BMI of 25–30 kg/m^2^ [22]. They reported lower body image and more symptoms of fatigue. High BMI was reported to be associated with improved sexual activity in women with vulvar cancer, with the hypothesis that estrogen production in adipose tissue might be related to improved sexual functioning [31]. This finding should be interpreted with caution as only 13 women with vulvar cancer were included in that study. Possible reasons for the lack of associations between BMI and quality of life in our study might be due to the small sample size. However, further research is necessary to further assess this association.

Despite our study not showing an association between BMI and lymphoedema, other studies have reported this. A previous study by Cirik et al. showed that obese women reported leg lymphoedema almost four times more than their non-obese counterparts after groin node dissection for vulvar cancer [32]. However, Cirik et al. measured the presence of lymphoedema within 1 year after surgery, while, in our study, the majority of patients were more than 5 years post-surgery. In other cancer areas such as endometrial cancer, the association between a higher BMI and the incidence of lymphoedema has also been reported [33,34].

In our study population, 17 (34.7%) participants were obese and 20 (40.8%) had a sedentary lifestyle. Interestingly, there was an equal distribution of physical activity levels among non-obese and obese participants. Van de Berg et al. previously reviewed the physical activity of vulvar cancer survivors and compared this to the general population. They reported a significant decline in physical activity after vulvar cancer surgery, with only 34.2% adhering to national guidelines [35]. These results are confirmed by other studies on gynecological cancer patients [24,36]. Sedentary lifestyle is an increasing problem in the UK, with around 30% of the total population being considered inactive [37]. In the primary care setting, 80% of consultants are unfamiliar with national physical activity guidelines, and at least 12% showed they were uncomfortable in raising the matter in consultations [38]. The high prevalence of sedentary behavior and the lack of knowledge of healthcare professionals indicate a need for better education to improve general health.

This is the first study to focus on vulvar cancer survivors that assesses the association between quality of life and lifestyle factors. Strengths included the use of validated questionnaires and a long follow-up time, and, therefore, taking into account the possible late effects of treatment [39]. The EORTC has, however, not yet validated the VU-34 vulva-specific module, which might change future interpretations. The study was limited by a small sample size, despite the long inclusion period. We believe this is due to the rarity of the disease and its presentation at a later age, with 45% of women deceased at the time of the survey. The response rate was 41.7%, with similar studies reporting response rates from 30 to 70% [39,40]. In addition, we used self-reported height and weight and exercise, which may not accurately reflect actual BMI and physical activity levels [41,42]. It has been established that obese people tend to underestimate their BMI, which may cause bias in the classification of participants in non-obese or obese groups and underestimate the effect of BMI [43]. Lastly, we did not have data on adjuvant treatment such as radiotherapy. The literature has shown that pelvic radiotherapy has a substantial influence on the quality of life after treatment, and women often experience long-term negative effects from this in other gynecological cancers [44,45]. We recommend that this be considered in future studies.

Future research would preferably be of a prospective nature. This would allow the observation of possible changes of quality of life, physical activity, and other lifestyle factors over time and to further delineate the cause–effect relationship. In addition, it would be interesting to see if lifestyle can be used as a modifiable factor to influence the quality of life of vulvar cancer survivors. As survivorship is often overlooked in vulvar cancer care, acquiring this knowledge will improve the current care and counselling that can be offered to these women. Physical activity programs and rehabilitation after treatment may then be offered to survivors with a sedentary lifestyle to improve their quality of life.

## 5. Conclusions

In this study, we showed that there is an association between physical activity and quality of life of vulvar cancer survivors, with increased physical activity levels being associated with improved overall quality of life and higher scores of physical, emotional, and cognitive functioning. In addition, sufficient physical activity was associated with lower symptom burden. Contrary to other gynecological cancers, we did not demonstrate an association between BMI and quality of life in vulvar cancer survivors. Future prospective research is therefore essential and should include the effects of adjuvant radiotherapy and possible changes over time. As most survivors are insufficiently active, it is important for rehabilitation opportunities to be provided to improve patient lifestyle and achieve improved quality of life and survivorship.

## Figures and Tables

**Table 1:** Baseline characteristics of participants and non-participants.

Clinical Characteristics	Respondents *n* = 58 (%/SD/IQR)	Non-Respondents *n* = 81 (%/SD/IQR)	*p*-Value
Age in years (mean, SD)	68 (10.7)	60 (13.6)	<0.001
BMI in kg/m^2^ at time of diagnosis (median, IQR)	28.5 (24.4–32.9)	29.0 (24.0–24.0)	n.s.
Missing value	8	18	
BMI categories			n.s.
<18.5 kg/m^2^	2 (3.4%)	1 (1.2%)	
18.5–24.9 kg/m^2^	11 (19.0%)	19 (23.5%)	
25.0–29.9 kg/m^2^	15 (25.9%)	15 (18.5%)	
≥30 kg/m^2^	24 (41.4%)	28 (34.6%)	
Missing value	6 (10.3%)	18 (22.2%)	
Primary vulvar surgery			n.s.
Wide local excision	31 (53.4%)	33 (40.7%)	
Vulvectomy	23 (39.7%)	43 (53.1%)	
Anovulvectomy	3 (5.2%)	4 (4.9%)	
Vulvovaginectomy	1 (1.7%)	1 (1.2%)	
Lymphadenectomy			n.s.
Inguinofemoral nodes	44 (75.9%)	57 (70.4%)	
Sentinel nodes	8 (13.8%)	21 (25.9%)	
None	6 (10.3%)	3 (3.7%)	
Tumor origin			n.s.
HPV	19 (32.8%)	27 (33.3%)	
Lichen sclerosis	29 (50.0%)	40 (49.4%)	
VIN NOS	7 (12.1%)	10 (12.3%)	
Bartholin’s gland	1 (1.7%)	1 (1.2%)	
Unknown	2 (3.4%)	3 (3.7%)	
FIGO stage			n.s.
IB	43 (74.1%)	67 (82.7%)	
II	3 (5.2%)	1 (1.2%)	
III	12 (20.7%)	13 (16.3%)	
Time since operation in months	64.6 (31.0–95.0)	70.1 (34.0–96.0)	n.s.
Recurrence			n.s.
Yes	12 (20.7%)	10 (12.3%)	
No	46 (79.3%)	71 (87.7%)	
Smoking at time of diagnosis			n.s.
Yes	9 (15.5%)	20 (24.7%)	
No	38 (65.5%)	41 (50.6%)	
Unknown	11 (19.0%)	20 (24.7%)	
ASA score			n.s.
I	3 (5.2%)	3 (3.7%)	
II	24 (41.4%)	36 (44.4%)	
III	9 (15.5%)	10 (12.3%)	
IV	1 (1.7%)	0 (0.0%)	
Unknown	21 (36.2%)	32 (39.5%)	
Comorbidities			
Cardiovascular disease	17 (30.4%)	32 (38.6%)	n.s.
Diabetes mellitus	10 (17.2%)	13 (16.0%)	n.s.
Respiratory disease	13 (22.4%)	14 (17.2%)	n.s.
Mental health issues	7 (12.1%)	10 (12.3%)	n.s.
Rheumatic/musculoskeletal diseases	10 (17.2%)	13 (16.0%)	n.s.

*n* = number of patients, SD = standard deviation, IQR = interquartile range, BMI = body mass index, HPV = Human Papilloma Virus, VIN = Vulvar Intraepithelial Neoplasia, NOS = not otherwise specified, FIGO = International Federation of Gynecology and Obstetrics, ASA = American Society of Anesthesiologists score. n.s. = non-significant.

**Table 2:** Median (IQR) of the outcomes of the EORTC-C30 and VU-34 questionnaires for non-obese and obese participants.

Quality Of Life Outcomes	Total Population *n* = 58 (IQR)	Non-Obese *n* = 32 (IQR)	Obese *n* = 17 (IQR)	*p*-Value Univariate Analysis
Global health score	66.7 (50.0–83.3)	62.5 (50.0–83.3)	66.7 (66.7–83.3)	n.s.
**Functional scales**				
Physical functioning	74.2 (46.7–93.3)	81.7 (53.3–96.65)	80.0 (60.0–93.3)	n.s.
Role functioning	83.3 (50.0–100)	66.7 (50.0–100)	83.3 (66.7–100)	n.s.
Emotional functioning	83.3 (66.7–100)	83.3 (66.7–100)	83.3 (58.3–100)	n.s.
Cognitive functioning	83.3 (66.7–100)	83.3 (66.7–100)	83.3 (66.7–100)	n.s.
Social functioning	83.3 (50.0–100)	91.7 (58.3–100)	100 (66.7–100)	n.s.
**Symptom scales**				
Fatigue	33.3 (11.1–55.5)	33.3 (11.1–55.5)	33.3 (11.1–44.4)	n.s.
Nausea and vomiting	0 (0–16.6)	0 (0–16.7)	0 (0–0)	n.s.
Pain	0 (0–33.3)	0 (0–33.3)	0 (0–33.3)	n.s
Dyspnea	0 (0–33.3)	0 (0–33.3)	0 (0–33.3)	n.s.
Insomnia	33.3 (0–33.3)	33.3 (0–33.3)	0 (0–33.3)	n.s.
Appetite loss	0 (0–33.3)	0 (0–33.3)	0 (0–16.7)	n.s.
Constipation	0 (0–33.3)	0 (0–33.3)	16.7 (0–33.3)	n.s.
Diarrhea	0 (0–33.3)	0 (0–33.3)	0 (0–0)	n.s.
Financial difficulties	0 (0–0)	0 (0–0)	0 (0–0)	n.s.
**VU-34 functional scales**				
Body image	66.7 (44.0–100)	77.8 (56.0–100)	44.0 (22.2–66.7)	0.043 *
Sexual enjoyment	58.3 (50–83.3)	50.0 (33.3–83.3)	66.7 (66.7–100)	n.s.
Sexually related vaginal changes	66.7 (33.3–88.8)	72.2 (66.7–100)	33.3 (0–66.7)	n.s.
**VU-34 symptom scales**				
Vulva skin changes	13.3 (0–20.0)	13.3 (0–20.0)	13.3 (6.7–40.0)	n.s.
Vulva scarring	16.7 (0–33.3)	16.7 (0–33.3)	16.7 (0–33.3)	n.s.
Vulvo-vaginal discharge	0 (0–0)	0 (0–0)	0 (0–0)	n.s.
Vulva swelling	0 (0–16.7)	0 (0–16.7)	0 (0–33.3)	n.s.
Groin lymphoedema	11.1 (0–22.2)	0 (0–11.1)	11.1 (0–33.3)	n.s.
Leg lymphoedema	20.8 (0–50.0)	8.3 (0–29.2)	50.0 (16.7–75.0)	0.006 *
Urine urgency and leakage	25 (8.3–50.0)	33.3 (8.3–50.0)	16.7 (8.3–41.6)	n.s.
Bowel urgency and leakage	16.7 (0–33.3)	16.7 (0–33.3)	0 (0–16.7)	n.s.

IQR = interquartile range, EORTC-C30 = European Organization for Research and Treatment of Cancer Core Quality of Life Questionnaire, VU-34 = vulvar cancer-specific module, n.s. = non-significant, * *p* < 0.05.

**Table 3:** Physical activity according to BMI groups.

	Non-Obese *n* = 32	Obese *n* = 17	*p*-Value
Sedentary lifestyle	13 (40.6%)	7 (41.2%)	n.s.
Moderately active lifestyle	6 (18.8%)	2 (11.8%)	
Active lifestyle	12 (37.5%)	5 (29.4%)	
Not disclosed	1 (3.1%)	3 (17.6%)	

n.s.= non-significant.

**Table 4:** Medians (IQRs) of EORTC-C30 VU-34 per activity level groups.

	Sedentary *n* = 25 (IQR)	Moderately Active *n* = 8 (IQR)	Active *n* = 18 (IQR)	Univariate *p*-Value	Multivariate *p*-Value
Global health score	50.0 (41.6–66.7)	75.0 (54.1–83.3)	83.3 (66.7–91.6)	<0.001 *	0.004 *
**Functional scales**					
Physical functioning	60.0 (46.7–73.3)	81.7 (46.7–91.1)	100 (86.7–100)	<0.001 *	<0.001 *
Role functioning	50.0 (33.3–75.0)	83.3 (33.3–100)	100 (100–100)	<0.001 *	<0.001 *
Emotional functioning	66.7 (50.0–83.3)	70.8 (41.6–91.6)	100 (88.8–100)	<0.001 *	<0.001 *
Cognitive functioning	83.3 (50.0–83.3)	91.6 (83.3–100)	100 (83.3–100)	<0.001 *	<0.001 *
Social functioning	66.7 (33.3–83.3)	83.3 (66.7–100)	100 (100–100)	0.001 *	0.003 *
**Symptom scales**					
Fatigue	33.3 (33.3–66.6)	22.2 (0–55.5)	11.1 (0–22.2)	<0.001 *	0.005 *
Nausea and vomiting	0 (0–16.6)	0 (0–0)	0 (0–0)	0.010 *	n.s.
Pain	0 (0–50.0)	24.6 (0–33.3)	0 (0–16.6)	n.s.	
Dyspnea	0 (0–33.3)	0 (0–33.3)	0 (0–0)	n.s.	
Insomnia	33.3 (0–33.3)	33.3 (16.6–50.0)	0 (0–33.3)	0.017 *	n.s.
Appetite loss	33.3 (0–33.3)	0 (0–33.3)	0 (0–0)	0.002 *	0.026 *
Constipation	0 (0–33.3)	16.7 (0–50.0)	0 (0–0)	n.s.	
Diarrhea	0 (0–33.3)	0 (0–0)	0 (0–0)	n.s.	
Financial difficulties	0 (0–33.3)	0 (0–0)	0 (0–0)	n.s.	
**VU-34 functional scales**					
Body image	66.7 (33.3–88.9)	56 (39.2–100)	77.8 (56–100)	n.s.	
Sexual enjoyment	83.3 (33.3–100)	50.0 (50.0–50.0)	58.3 (50.0–66.7)	n.s.	
Sexually related vaginal changes	77.7 (0–100)	66.7 (66.7–66.7)	66.7 (33.3–83.3)	n.s.	
**VU-34 symptom scales**					
Vulva skin changes	13.3 (6.7–40.0)	13.3 (0–26.7)	10 (0–20.0)	n.s.	
Vulva scarring	16.7 (0–50.0)	33.3 (0–33.3)	16.7 (0–33.3)	n.s.	
Vulvo-vaginal discharge	0 (0–0)	0 (0–0)	0 (0–0)	n.s.	
Vulva swelling	16.7 (0–33.3)	0 (0–0)	8.3 (0–33.3)	0.047 *	n.s.
Groin lymphoedema	11.1 (0–22.2)	0 (0–22.2)	5.5 (0–1.1)	n.s.	
Leg lymphoedema	25.0 (8.3–66.7)	25 (12.5–58.3)	12.5 (0–33.3)	n.s.	
Urine urgency and leakage	33.3 (20.8–45.8)	20.8 (0–50.0)	8.3 (0–50.0)	n.s.	
Bowel urgency and leakage	16.7 (0–33.3)	25.0 (0–50.0)	0 (0–16.7)	n.s.	

IQR = interquartile range, EORTC-C30 = European Organization for Research and Treatment of Cancer Core Quality of Life Questionnaire, VU-34 = vulvar cancer-specific module, n.s. = non-significant, * *p* < 0.05.

## Data Availability

Data will be available upon reasonable request.

## Abbreviations

HPV	Human Papilloma virus
BMI	Body Mass Index
FIGO	International Federation of Gynaecology and Obstetrics
ASA	American Society of Anesthesiologists
LSI	Leisure Score Index
EORTC	European Organization for Research and Treatment of Cancer
QLQ-C30	Core Quality of Life Questionnaire
VU-34	Vulva-specific Module of Core Quality of Life Questionnaire
IQR	Inter Quartile Range
SD	Standard Deviation
VIN	Vulvar Intraepithelial Neoplasia
NOS	Not Otherwise Specified
